# Elemental Composition and Implications on Brown Rice Flour Biofortified with Selenium

**DOI:** 10.3390/plants12081611

**Published:** 2023-04-10

**Authors:** Ana Coelho Marques, Fernando C. Lidon, Ana Rita F. Coelho, Cláudia Campos Pessoa, Diana Daccak, Inês Carmo Luís, Manuela Simões, Paula Scotti-Campos, Ana Sofia Almeida, Mauro Guerra, Roberta G. Leitão, Ana Bagulho, José Moreira, Maria F. Pessoa, Paulo Legoinha, José C. Ramalho, José N. Semedo, Lourenço Palha, Cátia Silva, Maria Manuela Silva, Karliana Oliveira, Isabel P. Pais, Fernando H. Reboredo

**Affiliations:** 1 Earth Sciences Department, Faculdade de Ciências e Tecnologia, Universidade Nova de Lisboa, Campus da Caparica, 2829-516 Caparica, Portugal; 2 GeoBioTec Research Center, Faculdade de Ciências e Tecnologia, Universidade Nova de Lisboa, Campus da Caparica, 2829-516 Caparica, Portugal; 3 Instituto Nacional de Investigação Agrária e Veterinária, I.P. (INIAV), Quinta do Marquês, Av. República, 2780-157 Oeiras, Portugal; 4 Instituto Nacional de Investigação Agrária e Veterinária, I.P. (INIAV), Estrada de Gil Vaz 6, 7351-901 Elvas, Portugal; 5 LIBPhys, Physics Department, Faculdade de Ciências e Tecnologia, Universidade Nova de Lisboa, Campus da Caparica, 2829-516 Caparica, Portugalrg.leitao@fct.unl.pt (R.G.L.); 6 PlantStress & Biodiversity Lab, Centro de Estudos Florestais (CEF), Associate Laboratory TERRA, Instituto Superior Agronomia (ISA), Universidade de Lisboa (ULisboa), Quinta do Marquês, Av. República, 2784-505 Oeiras, Portugal; 7 PlantStress & Biodiversity Lab, Centro de Estudos Florestais (CEF), Associate Laboratory TERRA, Instituto Superior Agronomia (ISA), Universidade de Lisboa (ULisboa), Quinta do Marquês, Av. República, Tapada da Ajuda, 1349-017 Lisboa, Portugal; 8 Centro de Competências do Arroz (COTARROZ), 2120-014 Salvaterra de Magos, Portugalcatia.leonardo.silva@gmail.com (C.S.)

**Keywords:** rice varieties, selenium biofortification, sodium selenate, sodium selenite

## Abstract

Rice (*Oryza sativa* L.) is one of the most economically and socially important cereals in the world. Several strategies such as biofortification have been developed in a way eco-friendly and sustainable to enhance crop productivity. This study implemented an agronomic itinerary in Ariete and Ceres rice varieties in experimental fields using the foliar application of selenium (Se) to increase rice nutritional value. At strategic phases of the plant’s development (at the end of booting, anthesis, and at the milky grain stage), they were sprayed with sodium selenate (Na_2_SeO_4_) and sodium selenite (Na_2_SeO_3_). In the first foliar application plants were sprayed with 500 g Se·ha^−1^ and in the remaining two foliar applications were sprayed with 300 g Se·ha^−1^. The effects of Se in the level of micro and macronutrients in brown grains, the localization of Se in these grains, and the subsequent quality parameters such as colorimetric characteristics and total protein were considered. After grain harvesting, the application of selenite showed the highest enrichment in all grain with levels reaching 17.06 µg g^−1^ Se and 14.28 µg g^−1^ Se in Ariete and Ceres varieties, respectively. In the Ceres and Ariete varieties, biofortification significantly affected the K and P contents. Regarding Ca, a clear trend prevailed suggesting that Se antagonizes the uptake of it, while for the remaining elements in general (except Mn) no significant differences were noted. Protein content increased with selenite treatment in the Ariete variety but not in Ceres. Therefore, it was possible to conclude, without compromising quality, that there was an increase in the nutritional content of Se in brown rice grain.

## 1. Introduction

The growing demand for nutritionally enriched foods is of increasing relevance, and the development or optimization of differentiating products becomes essential. The agricultural food systems are essential in providing most of the nutrients and compounds for human welfare and health although it is well known that micronutrient deficiency affects *c.a.* 3 billion people worldwide [1].

Biofortification is linked with the biological enhancement of food cultures using for example macro and micronutrients through a conventional plant breeding approach, genetic engineering, or agronomic methodologies [1]. Thus, the use of the most-consumed crops to achieve such goals is fully justified in both developed and developing countries [2,3,4,5].

Rice (*Oryza sativa* L.) is one of the most economically and socially important cereals in the world [6,7]. For instance, the population of Bangladesh consume around 145 kg/year per person [8], while in Portugal, the consumption of white rice is around 14 kg/year [9]. White rice is the most consumed form, however, brown rice has a high nutritional value [10]. There is a loss of vitamins and certain nutrients in industrial processing due to the removal of the bran (polishing process). Additionally, some studies have found that in rice genotypes (*indica* and *tropical japonica*), frictional polishing (8–10% loss of grain weight) decreased the concentration of Manganese (Mn), Iron (Fe), Potassium (K), and Phosphorus (P) by 60–80% [11]. Nevertheless, the loss of vitamins and some nutrients varied according to the ease of polishing the genotypes. In smooth polishing a 3–5% weight loss can occur, while Zinc (Zn) and Calcium (Ca) decreased by around 30%, regardless of the polishing technique or ease of polishing [11].

Selenium (Se) deficiency affects approximately 0.5–1 billion population in the world [12]. Additionally, studies based on a daily rice consumption (300 g day^−1^) reported insufficient concentration of Se in 75% of the white grains [13]. Thus, strategies have been developed to apply Se in plants in the form of selenite, selenate, selenium organic compounds, and nano-selenium, although currently a promising innovation using natural plant biostimulants (PB) is used to ameliorate plant performance due to the synergistic action of different compounds to improve crop productivity and quality, nutrient efficiency and abiotic stress tolerance [14,15]. Among them we must indicate humic acids and seaweed extracts, complex organic materials, chemical elements such as Se, and Si, inorganic salts including phosphite, chitin and chitosan derivates, antitranspirants (kaolin and polyacrylamide), and free amino acids and N-containing substances [16,17], as well as beneficial arbuscular mycorrhizal fungi and N-fixing bacteria of strains belonging to the genera *Rhizobium*, *Azotobacter*, and *Azospirillum* [18].

Several ways of applying Se have been developed such as soil application, soaking or seed coating, and foliar fertilization [19]. Studies showed that in rice, foliar application of sodium selenite is more effective than sodium selenate [20]. This foliar spraying strategy is considered more efficient than soil application [21] because it can be applied at a certain stage of the plant development, thus favoring the uptake and the rapid assimilation. Furthermore, the root does not interfere with the translocation process to the aerial part and there are no losses by fixation, in this case of Se, in the soil [22]. It is also known that Se ions are transported by the phloem and xylem, diffusing easily into epidermal cells and thus becoming part of the plant [23]. Additionally, Se acts as a co-factor for antioxidant enzymes, for example glutathione peroxidase [24]. The absorption of Se by the plant depends, among other factors, on variety and growing conditions. Climate change such as temperature, rainfall, wind velocity, and relative humidity can have an important effect on crops [25] as well as the intrinsic characteristics of the soil, such as pH, organic matter content, and cation exchange capacity, among others, which influence the elemental composition of the soils and the availability to plant uptake [26]. 

Previous Se biofortification studies in non-polished rice grains of Ceres variety have shown that with the application of selenate and selenite (up to 100 g Se ha^−1^) it was possible to obtain 9.44 mg·kg^−1^ and 17.7 mg·kg^−1^ of Se increment, respectively [27], without the appearance of toxicity symptoms and interference with the concentration of the studied elements, although the levels of Zn in control grains in both treatments (selenate and selenite) and different concentrations (25, 50, 75 and 100 g Se ha^−1^) were always higher than grains exposed to Se. Moreover, the natural interaction between macro and micronutrients is well documented in the literature [28,29] which can be potentiated when the load of a particular element is artificially increased through biofortification. For example, the excess of Zn through biofortification can influence negatively the uptake of Mn and Fe [30]. Studies with biofortified wheat plants with Se have shown that micronutrients were more affected than macronutrients [31].

Characteristics such as yield and quality make the Ariete variety highly appreciated by farmers [32]. This variety, of Italian origin, is the most grown in Portugal (for about 30 years) [33]. Additionally, the Ceres variety was the first Portuguese variety of carolino introduced in the catalog of cultivars that result from crossbreeding performed under the National Program for Genetic Improvement of Rice. In common, both have the fact that their grains are classified as long with a ratio of grain length/width of 2.6 and 2.5, respectively [34]. 

Considering the national and international importance of rice, this study aims to promote the enrichment of rice grain, of Ariete and Ceres varieties, in Se and thus to develop an agronomic itinerary. In addition, the impact on macro or micronutrients, the distribution of Se in brown rice grains, and colorimetric parameters were also evaluated. Considering the importance of whole grain and flours, quality analyses such as colorimetric parameters and total protein quantification were also performed.

## 2. Materials and Methods

### 2.1. Experimental Fields

The trials were conducted at the experimental station of the Rice Technological Center (COTARROZ) located in Salvaterra de Magos, Portugal, to grow two rice varieties, Ariete and Ceres, which were tested. 

### 2.2. Selenium Biofortification

Biofortification was carried out by three foliar pulverization with solutions of sodium selenate (Na_2_SeO_4_) and sodium selenite (Na_2_SeO_3_) at the same concentration. Since in the 1st pulverization with 500 g Se·ha^−1^ on the end of booting visual toxicity symptoms were observed, this concentration was reduced to 300 g Se·ha^−1^ during anthesis and milky grain stage. Control plants were not sprayed at any time with Na_2_SeO_4_ or Na_2_SeO_3_. The randomized blocks and a factorial arrangement (3 concentrations × 2 forms of Se × 2 varieties × 4 replicates = 48 plots) with the plot size for each replication was 8 m length × 1.2 m width = 9.6 m^2^. The cultural techniques adopted in soil preparation and sowing were those traditionally used in rice cultivation in the region. The agronomic management of trials, namely the control of weeds, diseases, and insect pests, the application of nitrogen fertilizers, and the irrigation water were the typically used for rice crops. The trial duration occurred from 22 August to 23 October 2019. The analysis was performed in dehusked grains (brown rice) of both varieties.

**Figure 1:**
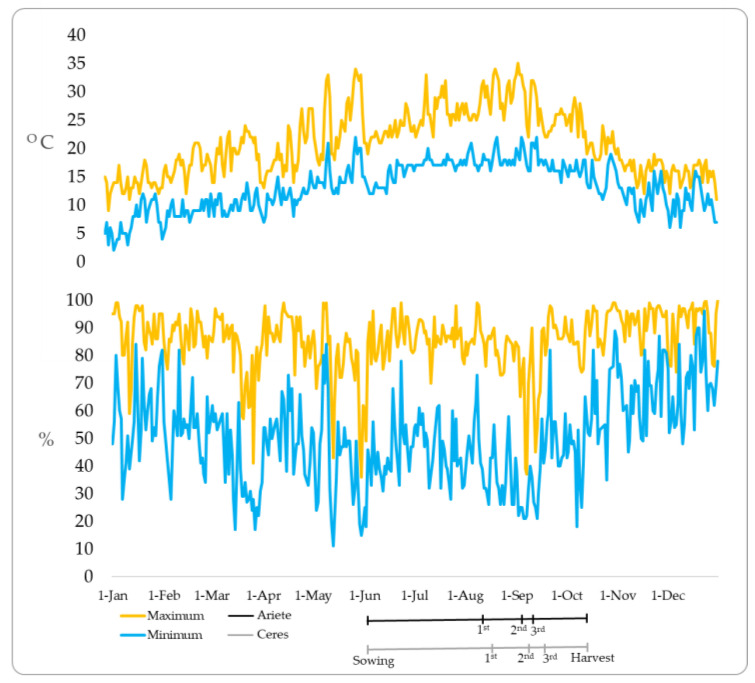
Maximum, minimum daily temperatures (°C), and the humidity (%) during the crop season (2019) at the weather station. The figure shows the day of sowing, foliar applications, and harvest in the rice experimental field.

The climate parameters associated with the rice production cycle in the COTARROZ field were measured by the weather station (38.957° N, 8.988° E). During the agricultural period, air temperatures reached a daily average of 17–26 °C. Additionally, the minimum and maximum recorded temperatures are 12 °C and 35 °C, respectively (Figure 1). During the rice growing cycle, the accumulated precipitation was 57 mm which corresponded to 64% relative humidity.

### 2.3. Quantification of Macro- and Micronutrients and Tissues Location of Selenium in the Brown Rice Grains

Quantification of Se, Zn, Mn, Fe, Ca, K, and P, and the localization of Se in brown grains, were determined in harvested grains from control and sprayed plots, with selenate and selenite, at 0 and 300 g Se·ha^−1^, using a µ-EDXRF system (M4 Tornado™, Bruker, Germany) according to Cardoso et al. and Reboredo et al. [35,36]. To enhance the ionization of low-Z elements, the X-ray generator was operated at 50 kV and 100 µA (without the use of filters). A set of filters between the X-ray tube and the sample, composed of three foils of Al/Ti/Cu, was used for a better quantification of Se and other medium-to-high atomic weight elements. Detection of fluorescence radiation was performed by an energy-dispersive silicon drift detector (XFlash™) with energy resolution of 142 eV for Mn Kα. The rice grain was cut, at the equatorial region, into slices with a stainless-steel surgical blade to better measure the distribution mapping of Se. Measurements were performed in the mapping mode and carried out under 20 mbar vacuum conditions. Additionally, by selecting the area corresponding to the entire grain, quantification analysis was performed on the obtained maps. Measurements were carried out in triplicate in the grains. Detection limits were the following: Ca = 35 mg kg^−1^; Fe = 6 mg kg^−1^; K = 60 mg kg^−1^; Mn = 9 mg kg^−1^; P = 2100 mg kg^−1^; Se = 3 mg kg^−1^; and Zn = 3 mg kg^−1^. Plant reference materials were used for data validation: orchard leaves (NBS 1571) and poplar leaves (GBW 07604); the recovery values ranged between 91 and 98%.

### 2.4. Colorimetry Analysis and Crude Protein Content

The colorimetric parameters of grain and flour samples were measured using a fixed wavelength according to the methods described elsewhere [37]. The system of the Commission Internationale d’Éclaire (CIE) was applied using the illuminant D65. Brightness/brightness (L) and chromaticity parameters (a* and b* coordinates) were obtained with a Minolta CR 300 colorimeter (Minolta Corp., Ramsey, NJ, USA) coupled to a sample vessel (CR-A504). The calibration was performed with the reference standard (L = 97.1, a* = 0.19, b* = 1.95). The mean values were obtained from four measurements per sample.

Crude protein in the whole flour was determined by the Kjeldahl method through quantification of total nitrogen considering that all nitrogen integrates the amino acid structure of proteins, according to Lidon et al. [38].

### 2.5. Statistical Analysis

Statistical analysis of the data was performed with the IBM SPSS Statistics 20 program, through a one-way analysis of variance and Tukey’s test for mean comparison considering a 95% confidence level. 

## 3. Results

### 3.1. Nutrients Accumulation in the Rice Grain

The yields of Ariete and Ceres varieties, in the selenate (Na_2_SeO_4_) treatment were 5749 and 6319 kg ha^−1^, respectively. However, in the selenite (Na_2_SeO_3_) application the yield of Ariete was 5400 kg ha^−1^ while in Ceres was 5903 kg ha^−1^. In the Ariete and Ceres variety controls, yields of 6024 and 6241 kg ha^−1^ were obtained, respectively.

**Figure 2:**
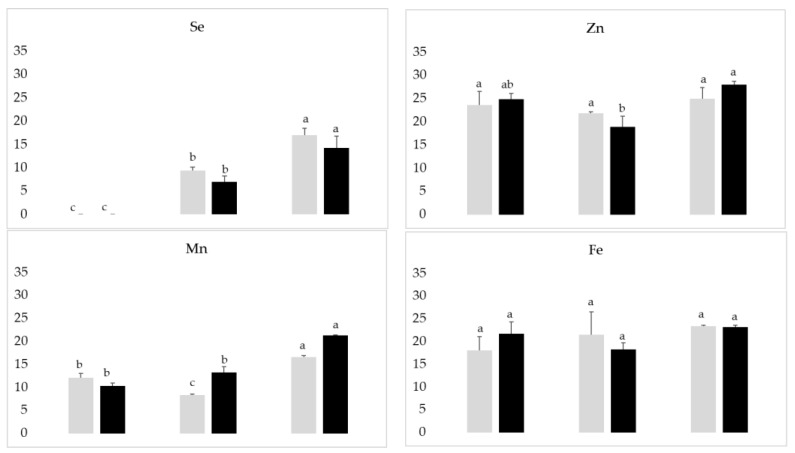

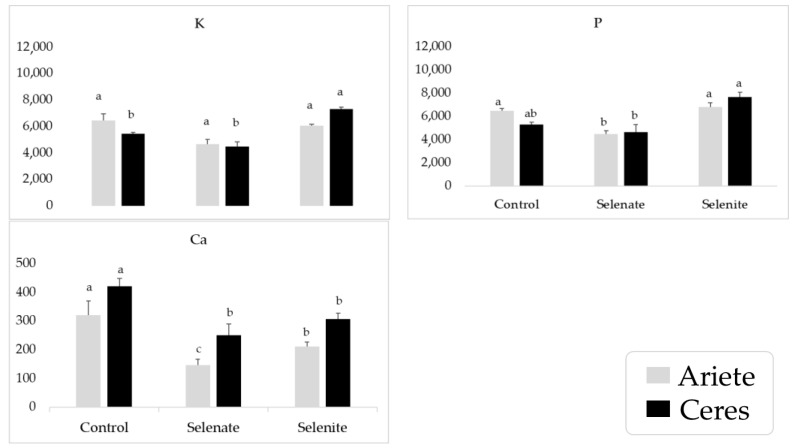
Average (µg g^−1^) ± standard error (*n* = 3) of Selenium, Zinc, Manganese, Iron, Potassium, Phosphorus, and Calcium concentrations in the brown rice grains. Letters a, b, and c indicate significant differences between treatments, for each variety (*p* ≤ 0.05).

The increase of Se in the brown grains of treated plants was observed, the highest levels being measured with the application of selenite, with a concentration of 17.06 µg g^−1^ in the Ariete variety and 14.28 µg g^−1^ in the Ceres variety (Figure 2). As such, the highest content obtained in Ariete is probably due to the variety capacity of accumulate Se. Regarding Zn level, the highest values were observed with the selenite treatment with a concentration of 24.94 µg g^−1^ in the Ariete variety and 28.03 µg g^−1^ in the Ceres variety, although the levels in general were not significantly different at the 0.05 significance level. Calcium decreases in both selenate and selenite treatments, in each variety. Thus, it seems that an antagonistic effect of Se on Ca uptake and/or translocation exists, although no data regarding the root system was available. 

Compared with control plants (6459 µg g^−1^) the decrease of P content of plants treated with Se was more pronounced after selenate application in both varieties (Figure 2) occurring the same regarding K levels. With selenite treatment the levels of K in the Ariete variety were close to the control levels, while in the Ceres variety treated plants had 1.3 times plus K than control plants. The highest concentration of Mn was verified in the Ariete variety (16.56 µg g^−1^) and Ceres (21.36 µg g^−1^), both of them after selenite treatment, while the levels of Fe were very close (*p* ≤ 0.05) regardless the varieties and treatments. In general, the content of both macro- and micronutrients was always higher when selenite was applied compared to selenate.

### 3.2. Selenium Localization in Rice Grains

**Figure 3:**
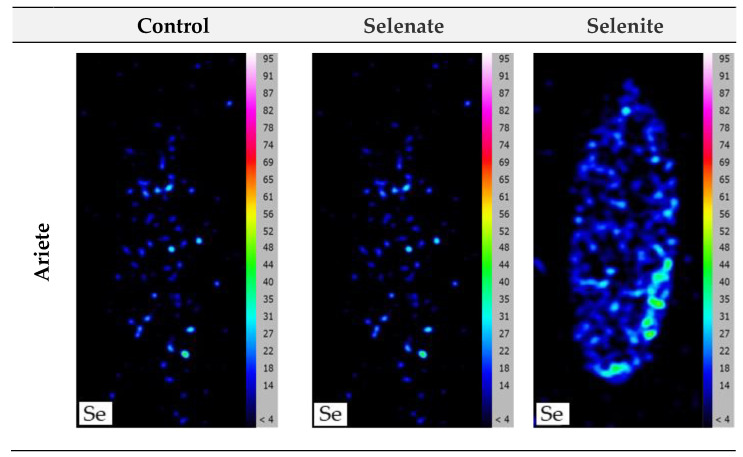

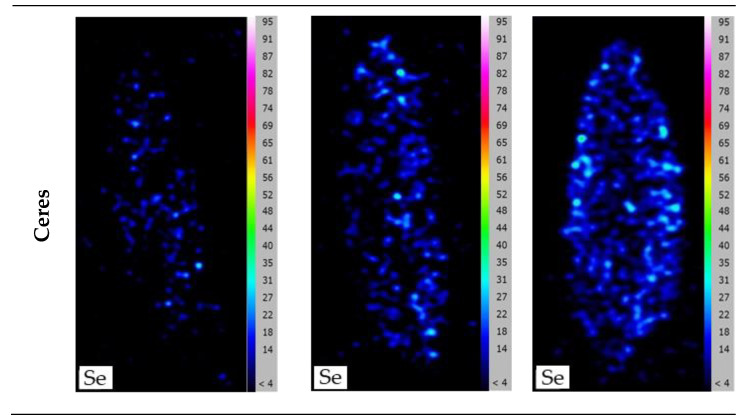
Localization of Se in brown rice grains of Ariete and Ceres varieties without receiving any type of Se enrichment (control) and after foliar pulverization with selenate and selenite (300 g Se·ha^−1^).

The location of Se was performed in brown rice grains after they were dehusked since the longitudinal cut removes the outer layer. This analysis showed that Se is evenly distributed throughout the grain, for both varieties (Figure 3). As stated before, the highest accumulation of Se was in the Ariete variety (17.06 µg g^−1^) after application with selenite.

### 3.3. Colorimetry Analysis

The colorimetric analysis performed on the grain and whole flour of the varieties under study revealed values higher than 50 for the brightness (L) parameter in both samples, although the L values were not significantly different. The highest values were observed in the flours compared to the grain (Table 1). In the grain, the highest L was 64.34, while in the flours it was 77.05, obtained in the selenate treatment (300 g·Se·ha^−1^) in Ariete and Ceres, respectively. Although without significant differences, the a* (red–green transitions) values were positive and low varying between 1.921–5.353 and 1.306–4.517 in the Ariete and Ceres varieties, respectively. This result revealed a tendency towards red color with less tendency in the whole flours. The positive values of b* (yellow—blue transitions) revealed a tendency towards yellow color, with the maximum value (25.55) registered in the grain of the Ariete variety. Although small oscillations were observed, there were no significant differences in the application of Se compared to the control.

### 3.4. Crude Protein Content

Total protein content was analyzed in whole flour of both varieties, with significant differences regarding the control (Table 2). There was a tendency to increase the total protein in the Ariete variety when applying the treatment with 300 g Se·ha^−1^ of selenite—the content increased from 6.323% to 6.902%. A significant decrease in total protein content of Ceres variety was observed when selenate was applied.

## 4. Discussion

The challenge of increasing the nutritional content of rice grains while respecting legislation and considering consumer requirements has disrupted the search for efficient solutions. The low content of Se in rice grains does not allow the requirements of the dietary intake of human populations to be met [39]. Rice biofortification is a promising strategy to address this problem by responding to nutritional imbalances spread worldwide. Nevertheless, the interactions between varieties and the environment can be a determinant to improving maximum productivity. 

Rice plants show the greatest sensitivity to high temperatures nine days before the panicle emerges (heading) and during this phase [25]. In our study, there was no heat wave or abnormal atmospheric phenomena (see Figure 1) interfering with normal plant development. Nevertheless, it is often essential to understand the effects of the changes in climate on the development of plants’ life cycle in both studies in vivo and in vitro [40,41,42]. Moreover, the increase of Se in rice grains depends on the form of Se used, the concentration tested, and the characteristics of each variety [6]. 

In our case, both varieties showed a significant increase in Se (Figure 2), particularly with the selenite treatment. These results agree with the literature since studies have reported that in rice, foliar application of sodium selenite is more effective than sodium selenate [20,27]. A previous study carried out with brown grains of Ariete variety, after foliar application of selenate and selenite at 300 g Se ha^−1^, showed a Se content of 13.17 mg kg^−1^ and 28.63 mg kg^−1^, respectively [43]. However, in the current work using the same variety and Se forms, the values ranged from 9.45 to 17.06 µg g^−1^. Although the grain is effectively biofortified, this discrepancy can be due to the interaction between the plant and environment in the paddy field, which is never uniform [44]. 

The results of selenate application in both varieties revealed Se contents ranging between 9.45 and 6.95 µg g^−1^ which shows similar assimilation capacity. In rice, probably because selenate is taken up via the sulphate transport system [45], it is more available for roots uptake than selenite [46]. However, due to the similar properties between selenate and sulfate, the uptake of this form of Se can be inhibited by sulfate because both share a common uptake mechanism—proton gradient sulfate transporters [47,48,49]. 

Additionally, regarding genotypic variability, under flooded conditions rice plants mainly take up selenite form [50] which is in agreement with our results. A study conducted with two genotypes (OP1505 and OP1509) that had been sprayed at different stages of rice development (booting, anthesis, and milky grain phase) with selenate and selenite in a range of concentrations (25, 50, 75, and 100 g Se ha^−1^) revealed the effectiveness of the biofortification process. Selenate treatment showed 4.9–7.1-fold increases in Se concentration in the grain while 5.9–8.4-fold increases were obtained with selenite treatments [51]. 

Independently of the foliar fertilization, the results of the localization analyses showed that the Se is evenly distributed throughout the grain in both varieties (Figure 3). This aspect is important because to obtain white grains the brown grain still must be processed (i.e., whitening), thus loosing part of its nutritional value. An X-ray fluorescence (µ-XRF) microanalysis of Se localization in rice seeds after foliar application, showed that Se was allocated in the endosperm in the form of hot spots [52]. In addition, studies in a mature rice grain slice using a synchrotron X-ray (XRF) revealed that Se is not only limited to the endosperm but extends to the aleurone/pericarp layer [13]. According to the literature, the main Se accumulation tissues are the endosperm, aleurone, embryo, and ovular vascular [38,39]. Thus, it is possible to obtain Ariete and Ceres white grains with high Se content even if to a lesser extent than brown grains.

Regarding the effects of Se application on the micro and macronutrient, studies have reported that after applying selenate and selenite to soil (0.75 mg·kg^−1^) the concentration of Fe in rice grains did not show significant differences, however, with foliar spraying (50 µm L^−1^) the highest levels were observed in selenate treatment [12]. In our study, the highest Fe levels were also recorded with selenate pulverization on the Ariete variety (Figure 2). Studies report that Zn concentrations in rice grains do not vary significantly with foliar spraying with Se [12]. In general, the levels of Fe and Zn were not influenced by Se application although in the case of Mn the highest levels were always observed in selenite treatments, regardless the varieties. Previous studies with rice show a tendency for inhibition of Zn accumulation when selenite is applied above 100 g Se ha^−1^ in both varieties [43], although the use of Se at 2 µM (Na_2_SeO_4_) in rice increases the Zn content in both plant parts [23].

A clear trend for a decrease in the Ca content in both selenite and selenate treatments, compared with control grains was noted, while in the case of K and P it seems that the treatment with selenate decreases the content of the grain, but not the selenite treatment (Figure 2). Calcium and potassium concentrations were higher in the bulbs of plants (*Allium cepa* L.) treated with both the selenium forms (selenocysteine and sodium selenate) than in the control plants [53], although the levels of P were not significantly different. The effects of selenite-Se at increasing concentrations (5, 25, 50, and 100 μmol⋅dm^−3^) on selected macronutrients of maize seedlings (*Zea mays* L. var. saccharata Kcke. cv. Zlota Karlowa) revealed that Ca and P content increased, while K content decreased with increasing Se treatments [54], thus showing the huge variability of the results mostly related with the experimental conditions and different plant species used.

There is a positive impact regarding color on consumer perception. Based on this, the quality parameters were considered. The parameter brightness (L) always revealed values >50 while a* and b* positive values (Table 1). Our data agree with studies concluding that further milling does not affect the brightness of rice [55]. Thus, selenium increment did not influence the colorimetric parameters of the flour and the grain, although external factors such as storage and drying can influence grain color. 

Compared to the white rice grain, the brown grain has a higher nutritional value because is constituted of bran layer, embryo, and endosperm, in the proportions of 6–7% *w*/*w*, 2–3% *w*/*w*, and ca. 90% *w*/*w*, respectively [56]. However, the whitening process eliminates tissues and consequently, the nutritional value of the grain decreases producing a by-product (rice bran) with high nutritional value [43]; however, the Se content is affected residually because it is distributed throughout the brown grain (Figure 3). Studies pointed out that the milling intensity contributes to decreasing nutritional quality and the content of P, K, Mg, Ca, and Mn of whole flour [57], because those elements were concentrated in the outer layer, but not Zn and Se which seem to be relatively evenly distributed in the grain, which agrees with Lidon et al. [43].

Although Fe content was not influenced by biofortification, it is known that most of the Fe is associated with the aleurone layer and embryo [58], both of which are eliminated in the whitening process, thus justifying the nutritional loss. High quantities of Zn (and Fe) are less concentrated in the endosperm because are bound to amino acids and free proteins in the bran and embryo [59]. Yet, associated with the increase of Se in the grains, the results seem to suggest, in the selenite treatment, a tendency to increase Mn, in the Ariete and Ceres varieties but a decrease in K and P in the same varieties treated with selenate. To characterize the rice nutritional quality, total protein is a key factor [60]. The application of selenite in the Ariete variety increased the total protein content (6.9%) (Table 2) and it was also in this treatment that the highest Se content was recorded (Figure 2), also contributing to the nutritional enrichment. Our findings are in accordance with the literature that showed a range of 4–10% of the protein in rice grains [60]. These results showed that more protein can contribute to the brown compounds in the surface coloring of the cookies, since Maillard reactions are promoted in the presence of more amino acids [61]. Whole flour of the Ceres variety has lower protein content and decreases when Se is applied in both forms compared to the control (Figure 2). Therefore, in products that aim for higher pasta viscosity, this flour will have greater applicability [23].

## 5. Conclusions

This study addressed an agronomic biofortification with selenate and selenite (300 g Se·ha^−1^) in Ariete and Ceres varieties, without reaching the toxicity threshold. Although both treatments had significant differences, it was in the selenite treatment that the brown grains showed the highest Se content. Although without significant differences in mineral content (except for P, K, and partially Mn), the data seem to suggest that selenite treatment can contribute to the increase of both macro and micronutrients. Since biofortification promoted the uniform distribution of Se in all grain tissues, it is possible to obtain brown and white grains of both varieties with high content of this micronutrient. The colorimetric parameters of the brown grain and whole flour were not affected by the applied biofortification, with the brightness remaining high. Although brown grain consumption is encouraged as it is a great nutritional source, after selenite application, the total protein content was also increased in the Ariete variety, thus highlighting its nutritional importance.

## Figures and Tables

**Table 1:** Average of color parameters ± standard error (*n* = 4) in brown rice grains and respective flours in Ariete and *Ceres varieties* L. (brightness), a* (red and green transitions) and b* (yellow and blue transitions).

Color Parameters				
Treatments		L	a*	b*
		Brown Grains		
Ariete	Control	61.53 ± 2.805 a ^1^	5.353 ± 0.882 a	25.55 ± 1.828 a
	Selenate	64.34 ± 1.921 a	4.506 ± 0.353 a	24.24 ± 0.389 a
	Selenite	61.94 ± 1.014 a	4.877 ± 0.163 a	24.00 ± 0.604 a
Ceres	Control	61.49 ± 3.314 a	3.047 ± 1.751 a	25.07 ± 1.701 a
	Selenate	58.43 ± 1.367 a	3.236 ± 0.808 a	23.30 ± 1.452 a
	Selenite	62.10 ± 1.339 a	4.517 ± 0.203 a	24.09 ± 0.510 a
		**Whole Flour**		
Ariete	Control	76.74 ± 1.837 a	2.031 ± 0.175 a	14.69 ± 0.473 a
	Selenate	75.79 ± 0.853 a	1.921 ± 0.107 a	14.35 ± 0.399 a
	Selenite	74.40 ± 1.261 a	2.217 ± 0.176 a	14.47 ± 0.590 a
Ceres	Control	76.68 ± 2.259 a	1.306 ± 0.358 a	15.12 ± 0.822 a
	Selenate	77.05 ± 1.288 a	1.657 ± 0.244 a	15.82 ± 0.189 a
	Selenite	71.99 ± 0.899 a	1.953 ± 0.177 a	15.09 ± 0.195 a

^1^ Letter a revealed the absence of significant differences among treatments, for each variety (*p* ≤ 0.05).

**Table 2:** Average (%) ± standard error (*n* = 4) of protein content in whole rice flour submitted to foliar pulverization of sodium selenate and sodium selenite.

Total Protein Content			
Treatments	Control	Selenate	Selenite
Ariete	6.323 ± 0.047 b ^1^	6.121 ± 0.034 b	6.902 ± 0.133 a
Ceres	6.113 ± 0.163 a	5.899 ± 0.069 b	5.983 ± 0.170 a

^1^ Letters a and b indicate significant differences between treatments, for each variety (*p* ≤ 0.05).
